# Knowledge, attitudes and practices of the medical personnel regarding atopic dermatitis in Yaoundé, Cameroon

**DOI:** 10.1186/s12895-017-0053-x

**Published:** 2017-02-16

**Authors:** Emmanuel Armand Kouotou, Jobert Richie N. Nansseu, Alexandra Dominique Ngangue Engome, Sandra Ayuk Tatah, Anne Cécile Zoung-Kanyi Bissek

**Affiliations:** 1 Department of Internal Medicine and Specialties, Faculty of Medicine and Biomedical Sciences, University of Yaoundé I, P.O. Box: 8314, Yaoundé, Cameroon; 2 Yaoundé University Teaching Hospital, Yaoundé, Cameroon; 3 Biyem-Assi District Hospital, Yaoundé, Cameroon; 4 Department of Public Health, Faculty of Medicine and Biomedical Sciences, University of Yaoundé I, Yaoundé, Cameroon; 5 Department of Paediatrics and Specialties, Faculty of Medicine and Biomedical Sciences, University of Yaoundé I, Yaoundé, Cameroon

**Keywords:** Atopic dermatitis, KAP study, Medical staff, Cameroon

## Abstract

**Background:**

Atopic dermatitis (AD) is a chronic, relapsing and pruritic inflammatory skin disease whose management remains unclear to most non-dermatologists. This study aimed to assess the knowledge, attitudes and practices (KAP) of the medical staff regarding AD in Yaoundé, Cameroon.

**Methods:**

This was a cross-sectional study conducted from January to April 2014 in 20 health facilities located in Yaoundé, the capital city of Cameroon. All medical staff who provided their consent were included in the study. A score was established for each of the KAP categories, and subsequently grouped into 4 classes considering a score <50, 50-<65, 65-<85 or ≥85%, respectively.

**Results:**

We enrolled 100 medical personnel, 62% of whom were females. Overall, the level of knowledge on AD was moderate (65%). Allergy was the main cause of AD, stated by 64% of participants. Only 43% personnel cited the genetic cause. Asthma was mentioned by 78% as an associated pathology. Regarding attitudes, the majority (84%) thought that AD is equally common among Black and Caucasian populations; 42% of participants believed that evolution is favorable when appropriate medical treatment is prescribed. These attitudes were considered wrong (64%). Similarly, the general level of practice was inadequate: 50%.

**Conclusion:**

Levels of knowledge, attitudes and practices of the medical staff regarding AD were poor, implying that management of this condition is non optimal in our setting.

**Electronic supplementary material:**

The online version of this article (doi:10.1186/s12895-017-0053-x) contains supplementary material, which is available to authorized users.

## Background

Atopic Dermatitis (AD) is a chronic, relapsing and pruritic inflammatory skin disease [1]. It is a condition that predominantly affects children especially of a certain age, in developed countries and increasingly in the developing world. An epidemiological study conducted at the Yaoundé General Hospital (Cameroon) by Zoung-Kanyi et al. found that allergic skin diseases were more common in children aged 0–5 years with AD as the leading one [2].

Onset of this condition occurs mostly in the first months of life. Indeed, 60–70% of cases start before the age of 6 months. Mesrati et al. in a pediatric dermatology unit in Tunisia noticed that 7.5% of consultations concerned AD [3], which contrasts with a prevalence of 10–25% reported in Western countries [4]. The young and immature immunity of the child underlies this increased susceptibility to outbreaks of AD, and consequently the high prevalence of AD in this age group. Indeed, AD is caused by defects in epidermal and cutaneous barriers which allow penetration of environmental molecules in contact with the skin. This results in a cutaneous inflammaxtion of which T-cells are responsible, directed against environmental allergens (extrinsic AD) and/or cutaneous auto-antigens (intrinsic AD). Moreover, AD can occur either in a context of atopy (predisposition to atopic states, known as extrinsic AD) or not (intrinsic AD) [5].

Evolution of the disease remains difficult to appreciate over years, although an improvement of signs is observed in regularly monitored patients. In fact, AD is usually characterized by surges and remissions at unpredictable frequencies, varying from one person to another with or without the influence of any driving factor, making it difficult to infer on the final issue. Following the exponential increase in AD in both developed and developing countries, restructuring of various aspects of its management has become essential to prevent complications.

But prior to this restructuring, an assessment of the knowledge and practices of the medical staff is mandatory, which will identify their weaknesses and subsequently enable improvement of their capacities. To the best of our knowledge, no study has already assessed the knowledge, attitudes and practices (KAP) of the medical personnel towards AD in Cameroon, a developing country. With the ultimate goal of improving the management of AD in Cameroonian hospitals, we undertook the present study which purposed to assess the KAP of medical professionals practicing in Yaoundé (Cameroon) with respect to AD.

## Methods

From January to April 2014, we conducted a cross-sectional study including the medical staff practicing in the city of Yaoundé. Five out of the seven existing health districts of Yaoundé were selected for this study and a total of 20 health facilities were visited. The choice of health districts and health facilities was arbitrary, taking into account the convenience for the investigators and accessibility of health facilities. The study population was comprised medical staff responsible for consultations in the selected health facilities: doctors (general practitioners and specialists/pediatricians) and nurses. At each visit to the study sites, all consulting staff irrespective of sex, seniority/experience, who consented to participate, were included. Our sampling was consecutive throughout the study period.

Before the study began, an ethical clearance was obtained from the Ethical Review Board of the Faculty of Medicine and Biomedical Sciences of the University of Yaoundé I, Cameroon. Authorizations were equally issued by health authorities of the selected health districts as well as directors of selected health facilities. The procedures were in compliance with the current revision of Helsinki Declaration. All aspects and procedures of the study were fully presented to each potential participant, and we included only those who voluntarily signed the consent form. Anonymity of participants and confidentiality of collected data were respected.

This study used as reference model the international consensus on management of AD [6]. An anonymous pre-tested and standardized questionnaire was used for data collection. All participants received the questionnaire to be filled; then the investigator returned on an appointed day to retrieve the questionnaire, after ensuring that this has been properly and extensively completed. The questionnaire, in addition to questions about age, gender, specialty and seniority in job/work experience, consisted of a set of 45 questions divided into three parts:

- Knowledge: theoretical clinical knowledge (primary lesions, localization, associated pathologies, causes); clinical knowledge based on recognition of iconography (Additional file 1); knowledge on prevention (information or advice to give to patients/parents in order to prevent an AD surge/relapse);
- Attitudes: perceptions of medical staff regarding the frequency of AD (specifically depending on race), evolution of AD with treatment, contribution of relatives in AD management and capacity of medical personnel to efficiently manage AD in our setting;
- Practice: number of cases of AD seen during consultations, prescription given for surges/relapses, route of administration of prescribed drugs, frequency of drug administration and monitoring of treatment.

The paper from Essi et al. on KAP studies [7] was used to establish scores for each part of the questionnaire. According to the sections (Knowledge, Attitudes, and Practice), the categories are distributed as follows:

- Knowledge: very poor (score <50%); poor (score: ≥50 and <65%); moderate (score: ≥65 and <85%); good (score ≥85%);
- Attitudes: harmful (score <50%); wrong (score: ≥50 and <65%); approximate (score: ≥65 and <85%); right (score ≥85%);
- Practices: harmful (score <50%); inadequate (score: ≥50 and <65%); average (score: ≥65 and <85%); adequate (score ≥85%).

Data were recorded and coded using Microsoft Excel 2007, then analyzed with SPSS v. 20 (IBM SPSS Inc., Chicago, Illinois, USA). Results are presented as frequency (percentage) for categorical variables and mean ± standard deviation (SD) for quantitative variables. To compare qualitative variables, we used the chi-square test. The level of statistical significance was set at *p* <0.05.

## Results

### Characteristics of the study population

We included 100 participants, predominantly females (62/100; 62.0%), giving a M/F sex ratio of 0.6/1. Our sample consisted of specialists (40/100; 40%), namely pediatric residents (20/100; 20%) and pediatricians (20/100; 20%), general practitioners (38/100, 38%) and State Registered Nurses (22/100; 22%).

## Knowledge

### General knowledge

#### Definition of AD

The majority of participants (75/100; 75%) were able to accurately define AD (Table 1).

**Table 1:** General knowledge of medical staff

Question	Right response Number (%)		Wrong response Number (%)	
General knowledge (*N* = 100)				
Definition				
AD is chronic and inflammatory	75	(75)	25	(25)
AD is chronic or inflammatory	7	(7)	93	(93)
AD is inflammatory and acute	13	(13)	87	(87)
AD is inflammatory or acute	2	(2)	98	(98)
I do not know	3	(3)	97	(97)
Associated pathologies				
Asthma is an associated pathology	78	(78)	22	(22)
Conjunctivitis is an associated pathology	58	(58)	42	(42)
Chronic cough is an associated pathology	16	(16)	84	(84)
There is no associated pathology	12	(12)	88	(88)
Causes				
The cause is psychological	2	(2)	98	(98)
The cause is allergic	64	(64)	36	(36)
The cause is genetic	43	(43)	57	(57)
The cause is infectious	18	(18)	82	(82)
I do not know	5	(5)	95	(95)
Evolution (*n* = 100)				
Evolution could be acute	54	(54)	46	(46)
Evolution could be chronic	77	(77)	23	(23)
Evolution is acute and chronic	46	(46)	54	(54)
Evolution is exclusively acute	1	(1)	99	(99)
Evolution is exclusively chronic	14	(14)	86	(86)

#### Associated pathologies

In our series, 78% (78/100) and 58% (58/100) respectively thought that asthma and conjunctivitis can occur in a patient with AD (Table 1).

#### Causes

Allergy was cited as the main cause of AD by 64% (64/100) of our participants, and genetics by 43% (43/100) (Table 1).

#### Evolution

Most participants 77% (77/100) described AD as a chronic disease; 54% (54/100) thought the condition is rather acute and 46% (46/100) thought it is both acute and chronic (Table 1).

## Theoretical clinical knowledge

### Primary lesions in AD

Concerning primary lesions of AD, the majority of health care providers cited xerosis cutis (86%; 86/100), erythema (81%; 81/100) and desquamation (58%; 58/100) as the main signs observed in AD (Table 2).

**Table 2:** Clinical theoretical knowledge as medical personnel

Question	Right response Number (%)		Wrong response Number (%)	
Theoritical clinical knowledge (*n* = 100)				
Primary lesions				
Xerosis cutis is a sign of AD	86	(86)	14	(14)
Erythema is a sign of AD	81	(81)	19	(19)
Desquamation is a sign of AD	58	(58)	42	(42)
Diffuse ulcerations is are signs of AD	17	(17)	83	(83)
Moist skin is a sign of AD	13	(13)	87	(87)
Cyanosis is a sign of AD	2	(2)	98	(98)
Sites in children (0–5 years old)				
The face is a site of AD	44	(44)	56	(56)
The torso is a site of AD	48	(48)	52	(52)
The lower limb is a site of AD	32	(32)	68	(68)
Sites in adults (25 years old and above)				
The face is a site of AD	24	(24)	76	(76)
The torso is a site of AD	42	(42)	58	(58)
The upper limb is a site of AD	79	(79)	21	(21)
The lower limb is a site of AD	41	(41)	59	(59)

### Sites in infants and young adults

In infants (0–5 years old), participants declared that the face (44/100; 44%) and torso (48/100; 48%) were the most likely localizations for AD (Table 2). On the other hand, the face (76/100; 76%) and trunk (58/100; 58%) were declared not to be privileged sites of AD in young adults (25–34 years old). Also, a little over half of participants (59/100; 59%) believed the lower limbs were a preferred site of AD lesions in adults (35 years old and above) (Table 2).

## Clinical knowledge based on iconography

Pictures 1 (80/100; 80%), 4 (75/100; 75%) and 5 (67/100; 67%) were selected for the diagnosis of AD while pictures 9 (15/100; 15%), 11 (15/100; 15%) and 13 (20/100; 20%) were not (Additional file 1).

## Prevention

The majority of participants (95/100; 95%) said they provide patients with advice and information about prevention. Furthermore, 91 (91) and 88 (88%) participants thought that cotton clothing are recommended and a relapse requires a new consultation, respectively.

## Attitudes

### Occurrence of AD based on race

AD is a disease that affects both Blacks and Caucasians alike according to 84% (84/100) of the medical staff.

### Opinion on evolution of AD

For 59% (59/100) of our sample, AD is a disease which evolves towards complete remission when patients receive the right treatment (Table 3).

**Table 3:** Attitudes of medical personnel

Question	Right response Number (%)		Wrong response Number (%)	
Opinion on occurrence of ad depending on race (*N* = 100)				
AD mostly affects Blacks	3	(3)	97	(97)
AD equally affects Caucasians and Blacks	84	(84)	16	(16)
No idea	13	(13)	87	(87)
Opinion on the evolution of ad with treatment (*N* = 100)				
Discouraging evolution	21	(21)	79	(79)
Favorable evolution	59	(59)	41	(41)
No idea	20	(20)	80	(80)
Opinion on contribution of relatives in management of ad (*n* = 100)				
Discouraging contribution	28	(28)	72	(72)
Favorable contribution	42	(42)	58	(58)
No idea	30	(30)	70	(70)
Opinion on ability to adequately manage ad in our setting (*N* = 100)				
AD can be managed	85	(85)	15	(15)
AD cannot be managed	7	(7)	93	(93)
No idea	8	(8)	92	(92)

### Patient care by the relatives

A total of 42 participants (42%) thought that patients could be taken care of by their relatives (Table 3).

### Management in our setting

In our series, 85% (85/100) thought that management of AD could be adequately provided in our setting (Table 3).

## Practice

Of the 100 participants, 91% (91/100) reported having encountered cases of AD during consultations. Table 4 shows their usual practice for every patient with this pathology.

**Table 4:** Practice of medical staff in case of AD relapses

Question	Right Response Number (%)
Number ad cases seen during consultations (*N* = 100)	
1-10 cases	51 (51)
11-20 cases	13 (13)
> 20 cases	25 (25)
No cases	11 (11)
^*^Drug prescription for ad relapse (*N* = 100)	
Corticosteroids	88 (88)
Antihistamines (AH)	64 (64)
Antifungal	14 (14)
Antibiotics	12 (12)
Corticosteroids + AH	56 (56)
Management of xerosis cutis: yes	81 (81)
Drug prescription in case of pruritus	
Antihistamines (AH)	90 (90)
Corticosteroids + AH	63 (63)
^*^Administration route of corticosteroids (*N* = 100)	
Oral	17 (20.2)
Topical	80 (96.4)
Oral + Topical	12 (70.6)
Administration modalities of topical corticosteroids (*n* = 88)	
1 application/day	38 (43.2)
2 applications/day	46 (52.3)
3 applications/day	4 (4.5)
Duration of the topical corticosteroid treatment (*N* = 100)	
< 2 weeks	43 (43)
2 weeks	30 (30)
1 month	12 (12)
> 1 month	0 (0)

^*^More than one answer was possible

Half of our sample (51/100; 51%) said they had received between 1 and 10 cases of AD per month at their consultations while 25% (25/100) reported having consulted more than 20 cases of AD.

Over half (79/100) of our sample chose to prescribe a medication for a patient with AD.

When a patient came to consult for AD, 88% (88/100) of the staff prescribed corticosteroids, most often topical (80/83; 96.4%) and to be applied 2 times a day (46/88; 52.3%).

### Management of xerosis cutis and pruritus

Xerosis cutis was supposed to be treated as declared by 81% (81/100) of the staff interviewed. For complaints of pruritus, 90% (90/100) of participants prescribed antihistamines and frequently associated antihistamines and corticosteroids, 63% (63/100) (Table 4).

## Association of knowledge, attitudes and practices of medical staff

### Score of knowledge among medical staff

The level of knowledge was conditioned by experience of the specialists. Indeed, more than half of pediatricians had a moderate level of knowledge (14/20; 70%), while 50% of residents had a poor level of knowledge (10/20; 50%). General practitioners had poor to moderate levels of knowledge about AD (Table 5).

**Table 5:** Scores of health care personnel according to the professional category

Consultant^*^	Scores^**^											
	very poor/harmful/harmful			poor/wrong/inadequate			moderate/approximate/average			good/right/adequate		
	K	A	P	K	A	P	K	A	P	K	A	P
Pediatricians	0 (0)	0 (0)	4 (20)	4 (20)	6 (30)	16 (80)	14 (70)	6 (30)	0 (0)	2 (10)	8 (40)	0 (0)
Residents	0 (0)	4 (20)	5 (25)	10 (50)	5 (25)	15 (75)	9 (45)	10 (50)	0 (0)	2 (5)	1 (5)	0 (0)
General practitioners	1 (2.6)	10 (26.3)	15 (39.5)	18 (47.4)	12 (31.6)	23 (60.5)	18 (47.4)	10 (26.3)	0 (0)	0 (0)	6 (15.8)	0 (0)
Nurses	2 (9.1)	2 (9.1)	9 (41.0)	11 (50)	2 (9.1)	12 (54.5)	9 (45)	7 (31.8)	1 (4.5)	1 (0)	11 (50)	0 (0)
Total	3 (3)	16 (16)	33 (33)	43 (43)	25 (25)	66 (66)	50 (50)	33 (33)	1 (1)	4 n	26 (26)	0 (0)

*Figures represent the number (percentage); **The scores were classified into: very poor/harmful/harmful = less than 50%; poor/wrong/inadequate = less than 65%; moderate/approximate/average = less than 85%; good/right/adequate = more than 85% correct answers; K = Knowledge; A = Attitude; P = Practice

### Score for attitudes among medical staff

Less than half (40/100) of pediatricians had a right attitude towards AD, Fifty percent of pediatric residents had an approximate score, General practitioners had approximate to harmful attitudes and nurses had approximate attitudes in relation to AD (Table 5).

### Score for practices among medical staff

Practices by all the professional categories were inadequate (Table 5).

After making an overall score for each parameter (knowledge, attitudes and practices), we observed that the medical staff of Yaoundé had a moderate level of knowledge (65%) with wrong attitudes (64%) and inadequate practices (50%) concerning AD.

## Discussion

This study on AD enabled us to assess the knowledge of medical professionals, their attitudes and clarify their different practices. A total of 100 subjects agreed to answer the questionnaire. Levels of knowledge, attitudes and practices were respectively 65%, 64% and 50%. It clearly appears thus that urgent measures need to be taken to strengthen our medical staff capacities in order to improve management of AD in Cameroonian hospital settings.

## Knowledge of the medical staff

Overall, most of the medical personnel had already heard about AD and was able to define it correctly. They were also able to recognize the characteristic lesions of the condition, probably as a result of their experience. The most frequently mentioned cause of AD was allergy (64%) in contrast to the genetic cause (43%). These results are significantly different from those found in the French national survey of professional practices on the treatment of AD [8]. In this survey, the genetic cause was indeed cited by almost all; regarding the allergic cause, two sources were cited by the different categories of professionals (allergists, dermatologists, general practitioners and pediatricians), namely food and inhaled allergens [8]. The psychological cause meanwhile was rarely mentioned by those participating in our survey, which mirrors findings from the French national survey indicating that 80% of physicians rarely or never suggested a psychological cause [8].

The medical staff demonstrated some confusion when asked about chronicity of AD because 77% of the staff declared that evolution could be chronic while 14% said it was exclusively chronic. Furthermore, half of our sample thought that this evolution could be acute. This significant confusion could be explained by the fact that in AD, the pruritic erythema is usually attributed to an acute pathology.

The medical staff had average knowledge on prevention. Pure cotton clothing was proposed by 90% of the staff as a preventive method. This choice of clothing is one of the recommendations of the 2005 consensus on the management of AD in children [6]. Most often, the staff believed a patient must return for consultation at every relapse of AD (87%) while 15% thought that the prescribed treatment would be sufficient to handle every relapse. Also, 63% of participants declared that moisturizing body lotions were prescribed for prevention. Certainly the 2005 consensus conference on the management of AD is in favor of keeping the skin moisturized permanently; however the medical professional must ensure that the chosen lotion or cream is conducive for treatment of xerosis cutis [6]. Furthermore 54% of our participants encouraged the use of antiseptic solutions for baths which is in contradiction with the 2013 consensus on AD, bolstering that the use of antiseptics for baths is indicated only for superinfected AD [9]. The moderate level of knowledge on AD in our sample (65%) already predicted an inappropriate management of the condition. This can be justified by the dearth of information on AD in our setting where it is considered a foreign pathology.

### Attitudes and practices of the medical staff

Half of our prescribers reported meeting between 1 and 10 cases of AD per month at consultations while only 25% had already received more than 20 cases/month. This result is contrary to those from the French survey where among various professional categories, 2 (pediatricians and allergists) saw more than 30 cases per month while only half of GPs (55%) saw less than 10 cases of AD per month [8]. AD is a disease that equally affects Blacks and Caucasians according to 84% of our sample, a somewhat contradictory result given that only 54% said they had not encountered many cases.

For 59% of our participants, AD usually has a favorable evolution when properly treated whereas 21% felt the outcome is actually unfavorable. Yet, AD classically involves intermittent periods of relapse and recovery even when properly treated. Overall, the staff had wrong attitude in relation to AD.

In case of AD relapses, our medical staff most frequently said they prescribe corticosteroids; 80% of these participants chose a topical corticosteroid, which is higher than those of the French study where dermatologists and general practitioners generally prescribed topical corticosteroid as first-line treatment in 60% and 28% of cases, respectively [8]. Moreover, in our study 56% reported prescribing a corticosteroid and an antihistamine in case of AD relapse but from the 2005 consensus conference on the treatment of AD, antihistamines have no place in the treatment of AD [6]. The 2013 consensus from the experts recommends the use of sedating antihistamines in case of intense pruritus [9].

Regarding the application of topical corticosteroid, half of medical staff recommended 2 applications per day while only 42% thought that the application of a topical corticosteroid once daily would be sufficient. According to Aubert et al., the use of corticosteroids differs depending on symptomatic variations in the patient and should be reasonable in order to avoid the risk of dependence or addiction [10]. Xerosis cutis is the sign in AD which prompted prescription of a treatment in 81% of our sample. Almost all prescribers said pruritus requires prescription of an antihistamine. Also, a combination of antihistamines and corticosteroids is systematic according to 63% of participants, although the 2005 consensus on AD states that administration of a topical corticosteroid alone would be effective because of its antipruritic and anti-inflammatory properties [6]. Clearly, the medical personnel adopted a poor practice in cases of AD most probably influenced by the moderate knowledge and inadequate attitude towards the condition.

The non-random selection of health facilities and the relatively small number of enrolled participants may constitute limits to the generalization of our results. Furthermore, many medical staff had not agreed to take part in this study and some were absent during our multiple visits to the facilities. Nevertheless, the use of the gradation developed by Essi et al. [7] allowed us to have a clear idea of the level of knowledge, attitudes and practices of our participants.

## Conclusion

This study allowed us to point out the moderate level of knowledge, wrong attitudes and inadequate practices of medical staff consulting in Yaoundé as far as AD is concerned, which suggests a poor quality of care delivered to patients with AD in our milieu, and a move towards emergence of complications. Management guidelines on this condition must be created and made available to our healthcare providers along with organization of regular continuous medical education sessions for them. Larger scale studies are needed throughout the country, to have a more precise mapping of the level of knowledge, attitudes and practices regarding AD in order to improve its management locally.

## Additional file

Additional file 1: According to you, is this a case of atopic dermatitis? Tick the correct answer. (DOCX 1417 kb)
