# Supplementary material for: Knowledge, attitudes and practices of the medical personnel regarding atopic dermatitis in Yaoundé, Cameroon
Source: BMC Dermatol. 2017 Feb 16;17:1. doi: 10.1186/s12895-017-0053-x (PMC5314472; doi:10.1186/s12895-017-0053-x)
Supplement: Additional file 1: — According to you, is this a case of atopic dermatitis? Tick the correct answer. (DOCX 1417 kb) [file 12895_2017_53_MOESM1_ESM.docx]

**Additional file 1**

**According to you, is this a case of atopic dermatitis? Tick the correct answer**


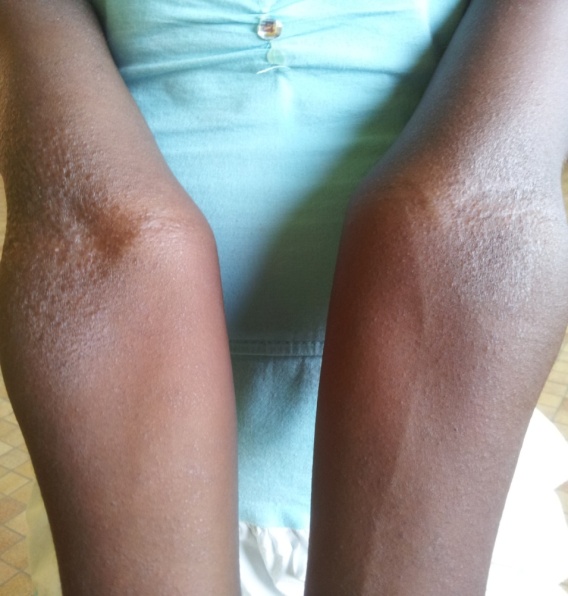

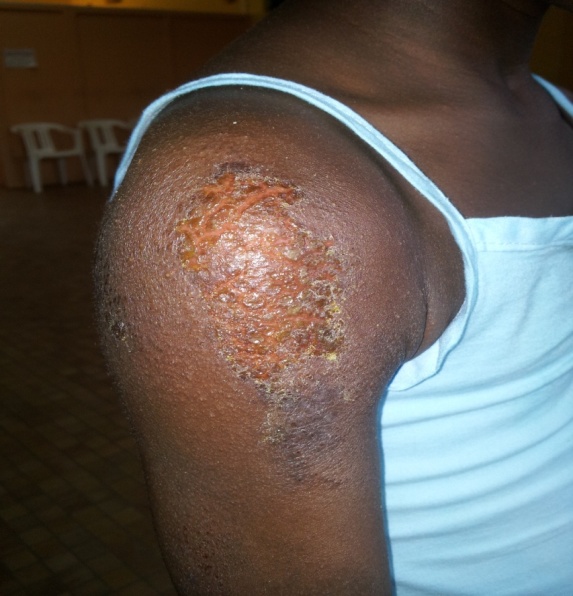

Picture 1:1=Yes  2= No Picture 2: 1=Yes  2= No


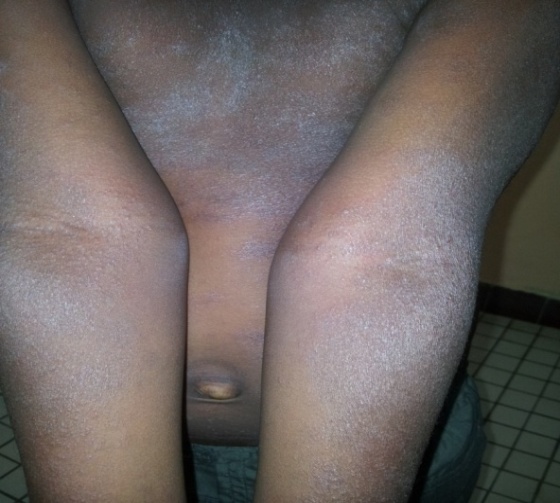

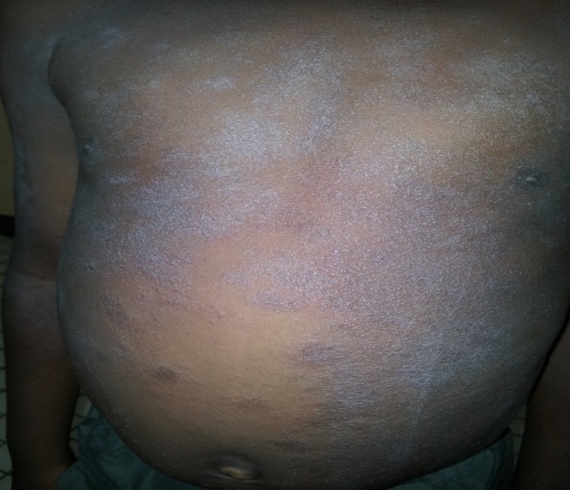


Picture 3: 1=Yes  2= No Picture 4: 1=Yes  2= No


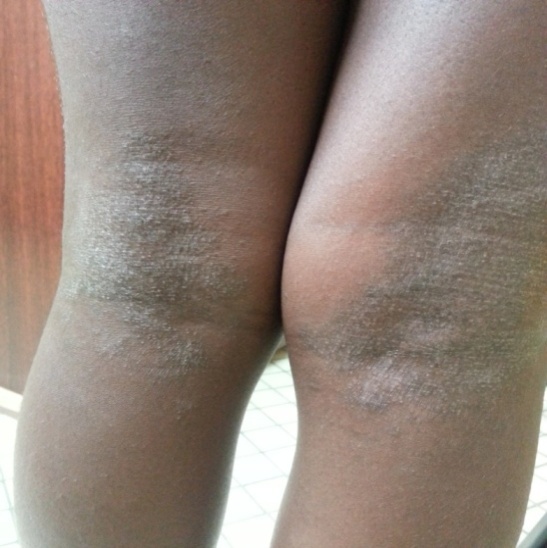

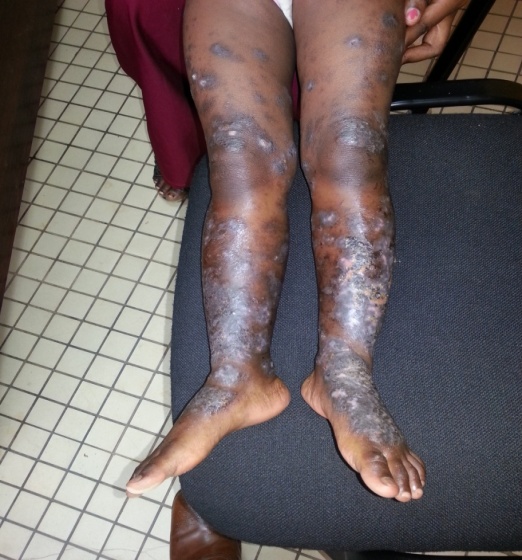


Picture 5: 1=Yes  2= No Picture 6: 1=Yes  2= No


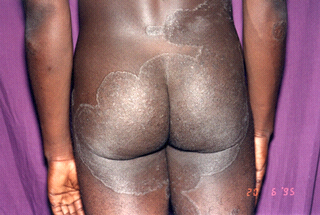

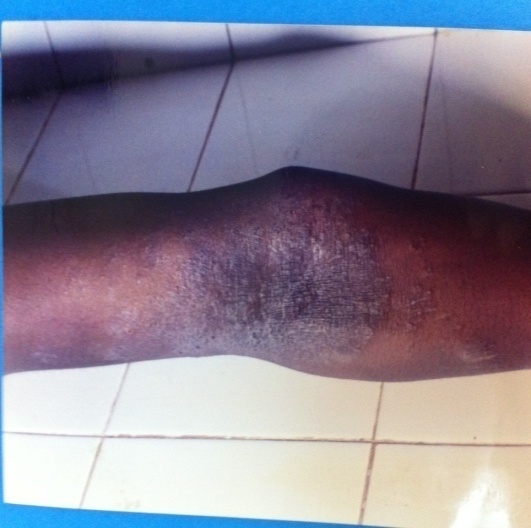


Picture 7: 1=Yes  2= No Picture 8: 1=Yes  2= No


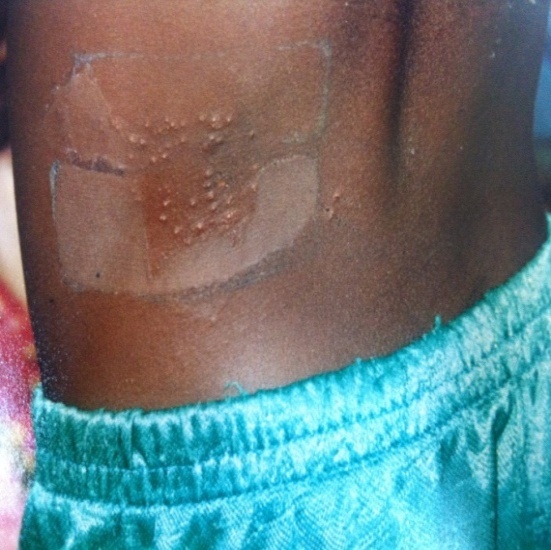

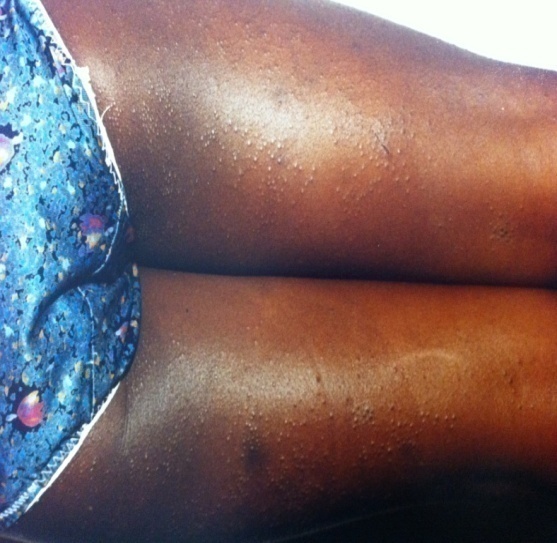


Picture 9 : 1=Yes  2= No Picture 10: 1=Yes  2= No


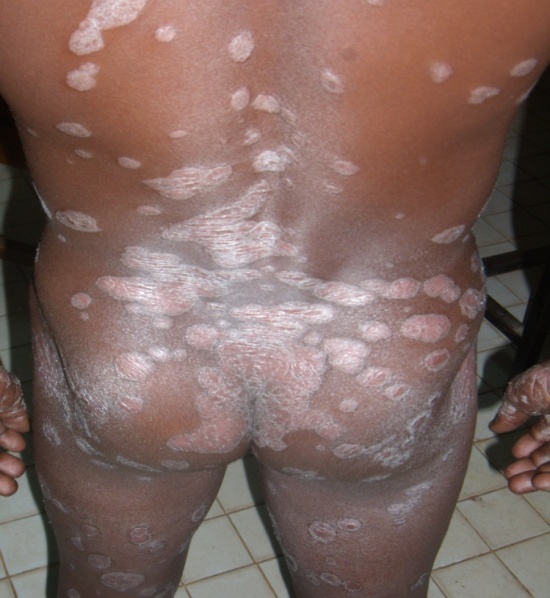

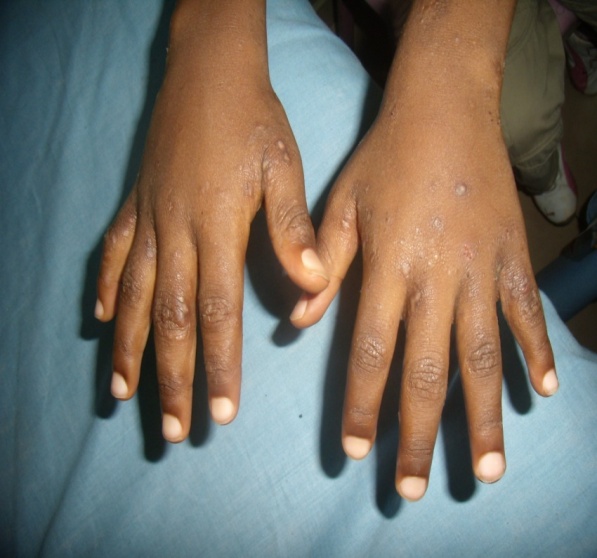


Picture 11: 1=Yes  2= No Picture 12: 1=Yes  2= No


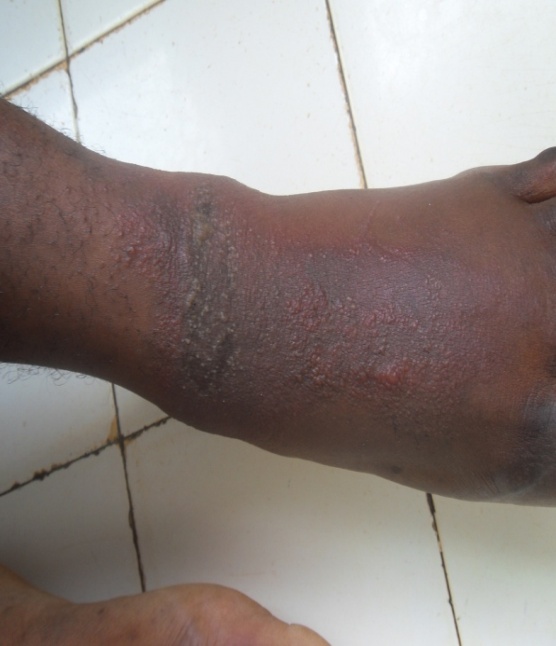

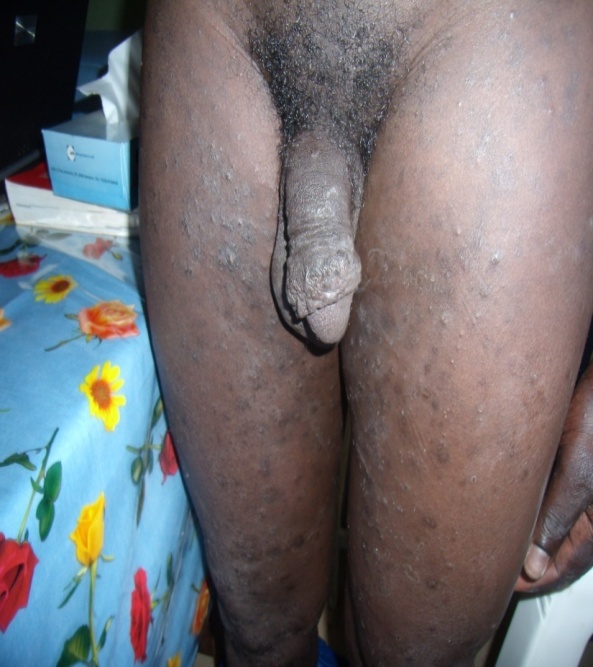


Picture 13: 1=Yes  2= No Picture 14: 1=Yes  2= No
